# Navigating nutritional information while living with metastatic cancer: a qualitative study of patient experiences and needs

**DOI:** 10.1007/s00520-026-11148-1

**Published:** 2026-09-02

**Authors:** Barbara S van der Meij, Manon GA van den Berg, Iris EC Nagtegaal, Floortje K Ploos van Amstel, Petronella B Ottevanger, Anja JThCM de Kruif

**Affiliations:** 1 Nutrition, Dietetics and Lifestyle, HAN University of Applied Sciences, Nijmegen, the Netherlands; 2 Department of Human Nutrition and Health, Wageningen University & Research, Wageningen, the Netherlands; 3 Department of Gastroenterology and Hepatology-Dietetics, Radboud University Medical Center, Nijmegen, the Netherlands; 4 Department of Medical Oncology, Radboud University Medical Center, Nijmegen, the Netherlands; 5 Department of Epidemiology and Datascience, Amsterdam UMC Location VUmc, Amsterdam, the Netherlands; 6 DeKruif Qualitative Research & Training, Niftrik, the Netherlands

**Keywords:** Metastatic cancer, Nutrition, Nutritional information, Patient experiences, Supportive care, Qualitative research

## Abstract

**Purpose:**

Nutrition often becomes a prominent concern for patients living with metastatic cancer. While patients frequently seek nutritional information to support their health and manage treatment-related challenges, they may encounter inconsistent advice from different sources. This study aimed to explore how patients with metastatic cancer perceive and navigate nutritional information, and to identify their experiences and needs regarding nutritional support during systemic therapy.

**Methods:**

A qualitative study using semi-structured interviews was conducted among patients with metastatic cancer receiving systemic therapy. Interviews were audio-recorded, transcribed verbatim and analyzed using reflexive thematic analysis.

**Results:**

Twenty-four patients (median age 55 y; 15 men; 9 women) with various tumor types participated. Three overarching themes were identified. First, nutrition became a more prominent concern after diagnosis, with patients reflecting on the role of nutrition during and after cancer treatment while adapting to treatment-related eating difficulties. Second, patients actively navigated nutrition-related information and advice from multiple sources, often encountering uncertainty and conflicting messages. Third, patients expressed a need for personalized, trustworthy and coordinated nutritional support. Respondents valued practical guidance tailored to their individual circumstances and preferred greater consistency between healthcare professionals.

**Conclusion:**

For many patients with metastatic cancer the challenge is not only obtaining nutritional information but also navigating and applying information from multiple sources in daily life. Nutritional support should therefore extend beyond information provision alone and include personalized, coordinated guidance that helps patients make informed decisions and manage nutritional concerns throughout treatment.

**Supplementary Information:**

The online version contains supplementary material available at https://doi.org/10.1007/s00520-026-11148-1.

## Introduction

Nutrition plays an important role in the treatment and well-being of patients with cancer. Adequate nutritional intake can support treatment tolerance and quality of life [1]. However, maintaining adequate nutrition during cancer treatment can be challenging due to symptoms such as decreased appetite, nausea, and changes in smell and taste, which may lead to reduced food intake and unintentional weight loss [1–3]. Nutritional counselling and nutrition support have been shown to help address these challenges and are therefore recommended in international guidelines, including those of the European Society for Clinical Nutrition and Metabolism (ESPEN) [1, 4].

In addition to questions about maintaining nutritional status, many patients are interested in the potential influence of dietary patterns and nutritional supplements on cancer outcomes [5]. However, evidence for many of these approaches remains limited, and clear guidance is often lacking [6]. As a result, healthcare professionals (HCPs) may experience uncertainty when discussing these topics with patients and may not always feel equipped to provide satisfactory answers [7].

When nutritional information needs are not adequately addressed, patients may seek information from a variety of other sources, including social media, online forums, fellow patients, and alternative practitioners [6]. However, information obtained from these sources is often inconsistent, incomplete or commercially driven [6, 8, 9]. This may lead to confusion, emotional distress, unnecessary dietary restrictions, financial burden, and the use of supplements with uncertain benefits or risks [9, 10]. In some cases, non-evidence-based advice may even interfere with treatment [11].

These challenges may be particularly relevant for patients living with metastatic cancer. Advances in treatment have enabled many patients to live longer with metastatic disease, often while receiving ongoing systemic treatment and experiencing a substantial symptom burden. In this context, nutrition becomes a recurring concern in daily life. Previous studies have shown that patients with metastatic cancer frequently expressed a strong need for nutritional information, particularly regarding symptom management and maintaining well-being [12–14]. Providing nutritional information that aligns with patients’ wishes and needs may help reduce uncertainty, support informed decision making, enhance well-being, and strengthen the patient–HCP relationship. Therefore, the aim of this study was to explore how patients with metastatic cancer perceive and navigate nutritional information, and to identify their experiences and needs regarding nutritional support during systemic therapy. This study forms part of a broader research project examining nutritional information needs and information provision in cancer care. A parallel study explored the perspectives of HCPs and is already reported in this journal [15].

## Methods

### Design and study population

An exploratory qualitative study using semi-structured interviews was conducted among patients diagnosed with metastatic cancer receiving systemic therapy at a university medical center. Inclusion criteria were age ≥ 18 years and ability to participate in a Dutch-language interview. Patients were excluded if they had cognitive impairments that limited their ability to understand the interview questions or provide reliable responses, such as documented dementia or severe cognitive decline. Patients were also excluded if physical conditions, such as severe fatigue or advanced illness, prevented them from participating in an interview. Eligibility was assessed by the treating physician or nurse based on their clinical judgment and knowledge of the patient's condition.

### Recruitment

Patients were recruited by oncology nurses working at the outpatient clinic. Purposive sampling was used to include variation in sex, age, tumor type, and systemic therapy, e.g. chemotherapy, immunotherapy, hormone therapy or targeted therapy [16]. After receiving verbal and written study information, patients provided written informed consent and an interview was scheduled.

### Interviews

Interviews were conducted face-to-face at the outpatient clinic by six trained student researchers (HAN University of Applied Sciences). Prior to data collection, student researchers received training in qualitative interviewing and were supervised throughout the study by two experienced qualitative researchers (AdK and BM). Interviews were audio-recorded using a digital voice recorder and reflective notes were taken after the interviews. Audio files were transcribed as soon as possible following the interviews and then removed from the recording device. The resulting transcripts including reflective notes were stored securely on the HAN University of Applied Sciences' protected research drive. Interviews took place between April 2022 and June 2023. An interview guide, based on literature and expert input (Supplementary file), included topics such as treatment-related eating difficulties, experiences with nutritional advice, and support needs. After the first few interviews, minor adjustments were made to the topic list to improve clarity and further explore topics raised in initial interviews.

### Data analysis

All interviews were transcribed verbatim by student researchers through manual transcription and subsequently anonymized. The analysis was conducted by two researchers with a background in nutrition and dietetics (BM, MB) and one experienced qualitative researcher (AdK). Data were analyzed in Microsoft Excel using reflexive thematic analysis (RTA) following Braun and Clarke [17, 18]. Analysis involved iterative phases of data familiarization, coding, theme development, reviewing, defining and reporting.

BM, MB and AdK first familiarized themselves with the data through repeated reading of the transcripts. Initial codes were generated manually and discussed in regular research meetings. Throughout the analysis, reflective notes were kept and codes, assumptions, interpretations and analytic memos were critically reviewed and refined through discussions among the research team, allowing reflection on alternative interpretations and promoting reflexivity.

Related codes were subsequently grouped into potential themes and organized into an initial thematic map. Themes were iteratively reviewed, refined, defined and named by BM, MB and AdK. Transcripts, codes and developing themes were continuously revisited and compared across interviews to explore similarities, differences and deviant cases. Alternative interpretations were actively explored and discussed within the research team following the principle of critical friends [19].

The multidisciplinary composition of the research team, including researchers with backgrounds in nutrition and dietetics and an experienced qualitative researcher, facilitated critical reflection on developing interpretations throughout the analytic process. Interpretations and assumptions were regularly discussed and challenged within the research team to promote reflexivity. Representative quotations were selected to illustrate the findings and ensure that respondents’ perspectives remained central to the presentation of the results.

### Ethical considerations

The study protocol was reviewed by the local human research ethics board, the Netherlands (2022) and deemed not subject to the Medical Research Involving Human Subjects Act (‘WMO’) (2022–13608).

## Results

### Patient characteristics

The study sample included men (*n* = 15; 62.5%) and women (*n* = 9; 37.5%) (median age: 55 y, range 25—78 y), representing a broad variation in tumour types. All patients had metastatic cancer and were on one or more systemic therapies, defined as chemotherapy (*n* = 21; 87.5%), immunotherapy (*n* = 8; 33.3%), hormone therapy (*n* = 8;33.3%), or targeted therapy (*n* = 2; 8.3%), sometimes combined with local treatments such as radiotherapy or surgery. Specific demographic characteristics are detailed in Table 1. Mean interview length was 48.7 ± 10.4 min.

**Table 1:** Demographics of 24 respondents with metastatic cancer

Variable	
*Sex (male:female), n (%)*	15 (62.5):9 (37.5)
*Age, median (range)*	55 (25–78) y
*Type of cancer, n (%)*	
*Breast cancer*	2 (8.3)
*Head and neck cancer*	4 (16.7)
*Colorectal cancer*	2 (8.3)
*Urinary tract cancer*	2 (8.4)
*Testicular cancer*	6 (25.0)
*Gynaecologic cancer*	2 (8.3)
*Pancreatic cancer*	2 (8.3)
*Bone cancer*	1 (4.2)
*Lung cancer*	1 (4.2)
*Melanoma*	1 (4.2)
*Thyroid cancer*	1 (4.2)
Treatment type n (%) *	
*Chemotherapy*	21 (87.5)
*Immunotherapy*	8 (33.3)
*Hormone therapy*	8 (33.3)
*Targeted therapy*	2 (8.3)
*Radiotherapy*	9 (37.5)
*Surgery*	14 (58.3)

^*^Some respondents received more than one oncological treatment

### Themes

The findings were organized into overarching themes, corresponding subthemes and nested subthemes (Table 2). Throughout the text, these are distinguished by formatting: themes are underlined, subthemes are presented in bold, and nested subthemes in italics.

**Table 2:** Themes, subthemes and nested subthemes identified from reflexive thematic analysis of interviews with 24 respondents with metastatic cancer

	*Theme*	*Subtheme*
1	Nutrition becomes a more prominent concern after diagnosis	a) Changing beliefs about nutrition and cancer • Awareness of nutrition's role in health and recovery • Views on nutrition and cancer development • Diet's perceived influence on treatment b) Living with nutrition-related challenges • Experiences with nutrition during treatment • Adapting eating practices • Finding a personal balance
2	Navigating nutrition-related information and advice	a) The search for suitable nutrition b) Obtaining and evaluating information c) Conflicting advice and self-management d) Interactions with healthcare professionals • Experiences with HCPs • Trust and uncertainty e) Influence of family, peers and social networks
3	Need for personalised and coordinated nutritional support	a) Personalised and trustworthy information b) Accessible and coordinated support c) Suggestions for improvement

The following three overarching themes were identified:

1. Nutrition becomes a more prominent concern after diagnosis
2. Navigating nutritional information and advice
3. Need for personalized and coordinated nutritional support

Theme 1 Nutrition becomes a more prominent concern after diagnosis

- Changing beliefs about nutrition and cancer

Following diagnosis, nutrition became a more prominent concern for many respondents. Respondents described becoming more aware of what they ate, reflecting on the possible role of nutrition in cancer development and treatment, and adapting their eating habits in response to treatment-related challenges. Nutrition became more important to most respondents after diagnosis:

“*Since I had cancer, I am more aware of what I eat…”* (Respondent 5)

#### Awareness of nutrition’s role in health and recovery

Patients acknowledged the importance of nutrition for recovery, health and longevity, emphasizing the need for making healthy and balanced choices:

*"I think there's a lot to be gained with nutrition and maybe you can live longer”.* (Respondent 4)

#### Views on nutrition and cancer development

Opinions about the influence of nutrition on the development of cancer were divided among patients. One patient believed that nutrition had no impact on the development of cancer, while others thought that healthy eating could reduce the risk of cancer:

*“Maybe I could have avoided getting cancer if I had known this about nutrition earlier”.* (Respondent 5)

Respondents indicated that they perceived a clear link between nutrition and the development of cancer and would like to know more about this. One respondent indicated that even though the oncologist did not recognize this relationship between nutrition and cancer, she was convinced nutrition plays an important role:

“*And the doctor said: you just had bad luck; this has nothing to do with your diet. You're living healthy, so… And yet I maintain that nutrition plays an important role in cancer. Yes, I would like to know more about that."* (Respondent 10)

#### Diet’s perceived influence on treatment

During cancer treatment, many respondents paid more attention to their diet. It was believed that consuming meat, sugars and alcohol could promote cancer growth and healthy eating and unprocessed foods, could inhibit it:

*"Sugar makes tumors grow, so I don't drink soft drinks anymore. Also, less alcohol."* (Respondent 5)

*"If I eat healthy, it's better to keep that tumor as small as possible…. yes… shrink or prevent it from growing."* (Respondent 8)

Some respondents stuck to their usual diet, with minor changes such as cutting out alcohol:

*"I eat the same as before, except no more alcohol."* (Respondent 15)

1. Living with nutritional challenges

### Experiences with nutrition during treatment

Systemic therapies such as chemotherapy often made eating difficult, especially due to taste and smell alterations:

*“I could no longer eat because my body refused to swallow anything that had a rotten taste in my mouth.”* (Respondent 3)

Eating could lose its social and emotional value:

*"Christmas is coming up. Well, we always had diner together. But now it will be a lunch "* (Respondent 12)

Some described eating as an exhausting necessity:

*“Food is the necessary evil.,,,[…]this chemical taste in your mouth, [,,] that just affects your smell and taste so much, that it just makes you really nauseous?"* (Respondent 9)

#### Adapting eating practices

Many respondents made practical adjustments to their diet to cope with treatment-related side effects, such as nausea, altered taste and smell, and reduced appetite. These adjustments, such as eating smaller meals, changing meal frequency, choosing milder foods or avoiding certain tastes and smells, were often based on advice from HCPs but were adapted by respondents to what was manageable in daily life. The environment in which meals took place was also important; eating at home often helped:

*"Eating at home is better than in the hospital. Like the other day: I came home and my son made a sandwich. Like a switch flipped again and I could eat and drink again."* (Respondent 9)

Simple remedies were helpful:

*"I feel weak after chemo. Tea with honey or fruit juice helps then."* (Respondent 16)

#### Finding a personal balance

Coping with nutritional challenges was seen as a personal process, shaped by physical changes, beliefs, and the level of support received. Some respondents mentioned that their attitude towards dietary advice changed over time:

*"I wasn’t open to dietary advice at all at first. But now I try to take them into account, even if I don’t always succeed."* (Respondent 15)

Respondents tried to find a balance between what was recommended and what felt manageable in daily life.

Theme 2 Navigating nutritional information and advice

Patients described an ongoing search for the “right” nutrition. While many actively sought information to support their health and treatment, they frequently encountered conflicting messages from HCPs, social networks, fellow patients and online sources.

- The search for suitable nutrition

The desire to understand what constituted the “right” food led many respondents into a continuous search for answers. They wanted to eat in a way that supported their health, but often encountered uncertainty and contradictory advice. Some had practical questions, such as whether certain foods could improve blood values or whether alcohol consumption was safe. The limited and sometimes inconsistent guidance from HCPs guidance left some respondents feeling responsible for figuring things out on their own:

“So many different people say something and have their own opinions. Then I’m just left on my own again.” (Respondent 2)

For some, this search was manageable; others described it as tiring or overwhelming, especially when they felt physically unwell or emotionally vulnerable.

2b) Obtaining and evaluating information about nutrition

Respondents differed in how they navigated nutritional information. Some relied on reliable sources:

*“You can find reliable information at KWF* [Dutch Cancer Society] *kanker.nl or dietetics websites*.” (Respondent 2)

Others avoided the internet because of conflicting opinions:

“*There is so much on the internet. I don't want to look at all that contradictory information.”* (Respondent 10)

Confidence in evaluating information varied:

*“I am highly educated and can think critically for myself: this is scientifically proven and this is nonsense.”* (Respondent 2)

“*Everyone gives advice. Facebook, fellow sufferers… this makes me doubt.”* (Respondent 6)

Some respondents actively explored natural or alternative approaches to support well-being:

*“Anything the alternative doctor can do to reduce my fatigue increases my quality of life.”* (Respondent 6)

Several respondents, were cautious, especially during immunotherapy:

*“I used to be open to trying things, but since I’ve been receiving immunotherapy and it’s working, I’m less keen on anything that might affect my immune system. I'm afraid it could interfere with the immunotherapy.”* (Respondent 13)

2c) Conflicting advice and self-management

Many respondents received differing or conflicting advice from HCPs, leading to uncertainty and the need to make their own decisions:

"*Advice from acquaintances, the GP, dietitians, and the radiation oncologist doesn’t match.*" (Respondent 2)

Perspectives from complementary and alternative caregivers could add to the confusion:

*"My acupuncturist often talks about intermittent fasting and cancer, and I'm not quite sure if I think that's wise or not."* (Respondent 2)

Many respondents experimented with diets, supplements or peer advice. For some this brought control; for others it led to doubt:

*"…. at a certain point, you no longer know what is and isn’t good"* (Respondent 6)

2d) Interactions with HCPs

### Experiences with HCPs

Most respondents were satisfied with the availability and approachability of HCPs and appreciated the support they received during treatment. Many described their interactions with nurses and physicians as accessible and reassuring:

*“They are always open to questions, so to speak. Even when we were at home. With questions you could just call, not a problem at all, and then they just speak to you politely****.**** Yes, I have to tell you that is excellent care, really. I am very happy with it.”* (Respondent 9)

Respondents also valued practical advice, although some strategies did not always relieve symptoms:

*"Ginger tea, flavored ice cream, custard was suggested, but they don’t always help."* (Respondent 10)

Support from dietitians, particular regular follow-up and personalized guidance was valued:

*"The dietician always called once a week or two weeks. If I have questions, I can call her back. "* (Respondent 16)

The growing awareness among HCPs about the role of lifestyle in cancer care was mentioned:

*"Doctors are now more open to nutrition and confirm that lifestyle matters."* (Respondent 11)

A smaller group of respondents felt that care focused mainly on treatment rather than prevention or broader wellbeing:

*"There is no prevention in the hospital, only treatment.* […….] *I really miss alternative medicine in the hospital, but I also understand it if it is not 100% sure." (*Respondent 5)

Some respondents expressed doubts about the nutritional knowledge of HCPs:

*"Most doctors are sincere, I don't doubt that, but they lack the knowledge about what nutrition can do."* (Respondent 11)

#### Trust and uncertainty

Some respondents emphasized their trust in their oncologist as their primary source of information. This trust was seen as vital, especially when it came to advice on supplements or diet during treatment:

*“I trust him, so to speak. What else can I do? Should I doubt the doctor?”* (Respondent 9)

While HCPs often asked about nutritional supplement use, concrete guidance was not always provided:

*"They also asked if I took supplements. Yes. Yes. But they didn't say I couldn't take them anymore. I had to find that out myself."* (Respondent 14)

2e) Influence of family, peers and social networks

Partners and family members supported respondents with shopping and meal preparation, especially when taste alterations were present. Without this support, respondents mentioned they would eat less or less healthy.

*“My wife also went completely crazy: what should I offer now? It has to be very fresh and very cold."* (Respondent 3)

In some cases, a partner was seen as the ‘nutrition expert’ in the household.

Sometimes household’ preferences caused tension or made it harder for respondents to make dietary changes:

“*I have three men in the house who want a piece of meat on the table every day….*.” (Respondent 10)

Friends or peers influenced choices:

*“Yeah, my little sister […], had a lot of trouble with her intestines and constipation and from her side I kind of get the tips …. like the Yakult that I just drank, that worked for her, so then I pick it up myself too.”* (Respondent 13)

Theme 3 Need for personalized and coordinated nutritional support

- Personalized and trustworthy information

Respondents expressed the need for clear, complete, accurate information:

*"I need to know that the information I receive is correct and current."* (Respondent 7)

Missing or unclear information from HCPs led to frustration:

*"Sometimes crucial information is missing, so I have to search elsewhere to find it."* (Respondent 3)

*"Sometimes I get conflicting information, and then I don't know what to believe."* (Respondent 15)

They also valued personal advice that was tailored to their specific situation.

*"Not all information is relevant to me. I would like it to be adapted to my needs."* (Respondent 9).

3b) Accessible and coordinated support

Respondents preferred information that was easy to understand and accessible, including visual formats:

*"I find it much easier to learn when there are images or videos."* (Respondent 5).

They also expressed preferences for different formats (paper versus digital) and valued the option of direct contact for complex questions.

3c) Suggestions for improvement

Many respondents shared ideas for how the information provision could be improved. They would like to receive relevant updates automatically, and in a way that matches their own needs and capabilities:

"*I don’t want to look up everything myself; I would prefer to receive updates."* (Respondent 19)

"*The information needs to be clearer; right now, it's sometimes too complicated*." (Respondent 21)

They also suggested developing joint nutritional guidelines that are easy to understand and supported by all involved HCPs.

They expressed a clear wish for more coordination:

*"It would be great if my GP, dietitian, and specialist worked together better so that I don’t get three different pieces of advice."* (Respondent 16)

## Discussion

This qualitative interview study explored how patients with metastatic cancer perceive and navigate nutritional information and to identify their experiences and needs regarding nutritional support during systemic therapy. Three overarching themes were identified. First, nutrition became a more prominent concern after diagnosis. Second, patients actively navigated nutritional information and advice from multiple sources, often encountering uncertainty and conflicting messages. Third, patients expressed a need for personalized and coordinated nutritional support.

A central finding of this study was that nutrition gained importance after diagnosis. Respondents described becoming more aware of their dietary habits and reflected on the possible role of nutrition in cancer development, treatment and recovery. At the same time, treatment-related symptoms such as altered taste and smell, nausea and reduced appetite often affected eating practices and changed the social and emotional meaning of food. For some respondents, eating became more difficult and less enjoyable, while others adapted their eating habits to accommodate treatment-related challenges. These findings suggest that nutrition in metastatic cancer extends beyond nutritional intake alone and is closely intertwined with daily routines, social relationships and quality of life, as previously reported in cancer populations [20]. Similar observations have been reported previously, with patients describing eating difficulties as having important psychosocial consequences that extend beyond physical nutritional intake [21].

Another important finding was the uncertainty patients experienced when seeking nutritional information. Respondents consulted a wide range of information sources, including HCPs, cancer organisations, online resources, family members, fellow patients, and complementary or alternative practitioners. While some respondents felt confident evaluating information, many encountered contradictory advice and struggled to determine which recommendations were trustworthy and applicable to their own situation. As a result, respondents often described feeling responsible for making decisions about nutrition themselves. Our findings suggest that patients are not only looking for more information, but also for support in navigating a complex landscape of nutritional information and advice.

Previous studies have shown that patients with cancer frequently hold beliefs about the relationship between nutrition and cancer and that nutritional information provided by HCPs may influence these [22]. However, our findings add nuance to this literature by suggesting that the challenge is not simply a lack of information. Rather, patients described having access to multiple sources of information, while simultaneously struggling with inconsistencies between these sources and uncertainty about their reliability and relevance. This suggests that the quality, consistency and applicability of nutritional information may be at least as important as its availability. Patients also expressed a broader interest in prevention, lifestyle and wellbeing in general, while HCPs focused on recommendations during cancer treatment.

The social environment also played an important role in this process. Family members, friends and fellow patients were frequently mentioned as sources of practical support, advice and encouragement. At the same time, these sources could contribute to uncertainty when their recommendations differed from those of HCPs. This highlights that nutritional decision making occurs within a broader social context and is influenced by interactions both inside and outside the healthcare setting.

A third key finding was the strong need for personalized, trustworthy and coordinated nutritional support. Respondents valued practical advice that took their individual situation, treatment and symptoms into account and expressed a clear preference for greater consistency between HCPs. The wish for better coordination was reflected in respondents’ desire to receive coherent nutritional advice across disciplines rather than having to reconcile differing recommendations themselves.

In the parallel study conducted within the same hospital, HCPs reported unclear role division and uncertainty particularly regarding nutritional supplements, underscoring the need for clearer responsibilities and improved support in nutritional care [15]. Similar barriers have also been reported in a national survey among HCPs in the United Kingdom, where respondents identified challenges in providing nutritional advice and support to patients with cancer [23]. Together, these findings suggest that improving nutritional communication requires not only patient education but also greater alignment, role clarification and collaboration among HCPs. Strengthening coordination between oncologists, nurses, dietitians and primary care professionals may help reduce contradictory messages and support the provision of consistent and patient-centred nutritional guidance.

### Strengths and limitations

A key strength of this study is its focus on patients with metastatic cancer, a relatively underrepresented population with many nutritional challenges which may remain relevant throughout prolonged treatment trajectories. By including respondents with different tumor types, ages, and various, sometimes combined, systemic treatments, this study captured a broad range of experiences and perspectives regarding nutrition and nutritional support. At the same time, the heterogeneity of the sample may have limited the exploration of differences between specific patient subgroups.

Another strength is the use of in-depth qualitative interviews, which enabled respondents to describe not only practical nutritional challenges but also the social, emotional and informational dimensions of nutrition during treatment. The RTA and the combination of expertise in nutrition and qualitative research facilitated critical reflection throughout the analytic process.

An additional strength is that this study formed part of a broader research project that also explored HCPs perspectives on nutritional information provision within the same hospital setting. The combination of patient and HCP perspectives allowed findings from both studies to be interpreted in relation to one another and provided a richer understanding of current nutritional care.

Several limitations should be considered. First, the study was conducted in a single university medical center, which may limit transferability to other healthcare settings. Second, participation was voluntary and may have attracted patients with a particular interest in nutrition, potentially resulting in selection bias. Third, although the interviews were conducted by trained student researchers under supervision of experienced qualitative researchers, differences in interviewing experience may have influenced the depth of probing in some interviews. However, students have no role in the regular healthcare process, which may also have contributed to a more open and equal interview. Finally, while the diversity of the sample enhanced the breadth of perspectives captured, the study was not designed to explore differences between tumour types, treatment modalities or demographic subgroups.

### Implications for practice

The findings of this study suggest that nutritional support in metastatic cancer care should extend beyond the provision of information alone. Patients frequently encountered nutritional information from multiple sources and often struggled to determine which advice was trustworthy and relevant to their personal situation. HCPs may therefore play an important role in helping patients interpret, contextualise and apply nutritional information throughout the treatment trajectory.

A more proactive approach to discussing nutrition may be beneficial, particularly during periods in which patients experience treatment-related symptoms or have questions regarding dietary changes, supplements or complementary approaches. Respondents valued practical, personalized advice that was tailored to their individual circumstances and treatment-related challenges.

The findings from this study, together with the parallel study among HCPs [15], highlight the importance of coordinated nutritional communication. Greater alignment between oncologists, nurses, dietitians and primary care professionals may help reduce contradictory messages and support the provision of consistent, patient-centred nutritional guidance.

## Conclusion

For many patients with metastatic cancer, nutrition becomes a more prominent concern after diagnosis and throughout treatment. Patients actively seek nutritional information from multiple sources but often encounter uncertainty due to inconsistent, incomplete or conflicting advice. As a result, the challenge appears to be not only obtaining information but also navigating and applying information in the context of daily life with cancer.

The findings of this study suggest that nutritional support should extend beyond the provision of information alone. Patients expressed a need for personalized, trustworthy and coordinated guidance that takes their individual circumstances, symptoms and treatment trajectory into account. Strengthening collaboration between HCPs and improving consistency in nutritional communication may help patients make informed decisions and feel more confident in managing nutrition during treatment.

## Supplementary Information

Below is the link to the electronic supplementary material.

Supplementary file1 (DOCX 38 KB)

## Data Availability

No datasets were generated or analysed during the current study.
