# Supplementary material for: Navigating nutritional information while living with metastatic cancer: a qualitative study of patient experiences and needs
Source: Support Care Cancer. 2026 Sep 2;34(9):924. doi: 10.1007/s00520-026-11148-1 (PMC13538155; doi:10.1007/s00520-026-11148-1)
Supplement: Supplementary file 1 [file 520_2026_11148_MOESM1_ESM.docx]

**Supplementary file - Interview guide**

Introduction

*Good morning/afternoon Mr./Mrs. [name],*
*My name is [interviewer’s name] and this is [interviewer’s name]. I will be interviewing you today. One of us will observe during the interview, keep track of time, and ensure that all topics are covered.*
*We are contributing to this research as part of our graduation project for the Nutrition & Dietetics and Nursing programs at HAN University of Applied Sciences in Nijmegen.*

*First, I would like to inform you once again about the purpose of this study and this interview. The goal is to gain insight into ideas and needs regarding nutrition in the context of cancer treatment.*

*With these results, we aim to help improve care for oncology patients in the future.*

*The interview will last approximately 45 minutes to one hour. If at any point you would like to take a short break or time-out, please feel free to let us know.*

*Before we start, do you have any questions about the patient information letter, or do you feel you are fully informed?*

*As you have read the information in the patient information letter, this interview will be recorded. As stated in the information letter, your data will be handled confidentially.*

Pause moment – answer any questions

*Do you have any further questions?*

*I would now like to move on to signing the informed consent form. By signing, you indicate that you have been properly informed about the study and that you give permission to participate. We will sign the form together. You may withdraw your consent at any time.*

**START THE RECORDING**

**MENTION THE PATIENT ID AT THE START OF THE INTERVIEW**

Opening question:

*How are you feeling today?*

Topics:

***Diagnosis / Treatment***

**Course of illness**

- *What is your diagnosis?*
- *How long have you had this diagnosis?*
- *What treatment(s) are you receiving or have you received?*

**Coping with the illness**

- *How do you personally experience the illness?*
- *How does your environment (family, friends, etc.) experience your illness?*

**General symptoms**

- *Which symptoms do you experience?*

***Nutrition and Illness***

**Complaints / side effects that affect eating or drinking**

- *What (specific foods) are affected?*
- *When (specific times or situations)?*
- *Have there been changes (eating differently)?*
- *Have you encountered any difficulties (e.g., limitations)?*

**Nutritional information received from healthcare providers**

- *Which healthcare provider(s) (e.g., physiotherapist, doctor, nurse, informal caregiver)?*
- *What kind of information did you receive?*
- *Why was this information given?*

**Engagement with nutrition**

- *To what extent are you concerned with nutrition?*
- *Why?*
- *How (in what way)?*
- *How important is it to you?*
- *How does making changes in your diet affect your daily life?*

**Role of nutrition**

- *In general*

**Role of nutrition in cancer**

- *What impact does it have (on life, home, friends, work)?*

**Questions about nutrition and cancer**

- *What questions do you have, and at what point during your treatment did they arise?*
- *What actions did you take to find answers (sought or asked for information, etc.)?*
- *Do you feel the need for more information? Are there gaps or areas for improvement — and in what way?*
- *What is your preferred way to receive information?*

**Herbs / supplements**

- *Have you researched or used them?*

***Support***

**Support experienced (from relatives and healthcare providers)**

- *In what way?*
- *From whom?*
- *Do you feel a lack of support or a need for more support?*
- *Interaction with fellow patients / peer support?*

***Conclusion***

- *I will now check with the observer: Have I asked everything? Have we covered all topics?*
- *We have gathered a lot of information. Is there anything else you would like to add or share?*

*Background information* (to check if anything has not yet been discussed):

- Age
- Gender
- Diagnosis
- Treatments (chemotherapy, immunotherapy, surgery, radiotherapy, other?)
- I*f any questions arise after this interview, may we contact you again?*
- *Would you be willing to participate in possible follow-up research later on?*
- *We would like to thank you very much for your time and participation in this interview.*
